# Moderate-intensity Combined Training Induces Lipidomic Changes in Individuals With Obesity and Type 2 Diabetes

**DOI:** 10.1210/clinem/dgae177

**Published:** 2024-03-15

**Authors:** Renata Garbellini Duft, Ivan Luiz Padilha Bonfante, Susana Alejandra Palma-Duran, Mara Patrícia Traina Chacon-Mikahil, Julian Leether Griffin, Cláudia Regina Cavaglieri

**Affiliations:** Department of Metabolism, Digestion & Reproduction, Imperial College London, London SW7 2AZ, UK; Laboratory of Exercise Physiology, Faculty of Physical Education, University of Campinas, 13083-851, São Paulo, Brazil; The Rowett Institute of Nutrition and Health, University of Aberdeen, Aberdeen AB25 2ZD, UK; Laboratory of Exercise Physiology, Faculty of Physical Education, University of Campinas, 13083-851, São Paulo, Brazil; Department of Metabolism, Digestion & Reproduction, Imperial College London, London SW7 2AZ, UK; Department of Food Science, Research Centre in Food and Development AC, Hermosillo, 83304, Mexico; Laboratory of Exercise Physiology, Faculty of Physical Education, University of Campinas, 13083-851, São Paulo, Brazil; Department of Metabolism, Digestion & Reproduction, Imperial College London, London SW7 2AZ, UK; The Rowett Institute of Nutrition and Health, University of Aberdeen, Aberdeen AB25 2ZD, UK; Laboratory of Exercise Physiology, Faculty of Physical Education, University of Campinas, 13083-851, São Paulo, Brazil

**Keywords:** exercise, obesity, diabetes, insulin resistance, lipidomics, mass spectrometry

## Abstract

**Context:**

Alterations in the lipid metabolism are linked to metabolic disorders such as insulin resistance (IR), obesity and type 2 diabetes (T2D). Regular exercise, particularly combined training (CT), is a well-known nonpharmacological treatment that combines aerobic (AT) and resistance (RT) training benefits. However, it is unclear whether moderate-intensity exercise without dietary intervention induces changes in lipid metabolism to promote a “healthy lipidome.”

**Objective:**

The study aimed to investigate the effect of 16 weeks of CT on plasma and white adipose tissue in both sexes, middle-aged individuals with normal weight, obesity (OB), and T2D using an ultra-high performance liquid chromatography–mass spectrometry (UHPLC-MS) untargeted lipidomics approach.

**Methods:**

Body composition, maximum oxygen consumption (VO_2_max), strength, and biochemical markers were evaluated before and after the control/training period and correlated with lipid changes. CT consisted of 8 to 10 RT exercises, followed by 35 minutes of AT (45%-70% VO_2_max), 3 times a week for 16 weeks.

**Results:**

The CT significantly reduced the levels of saturated and monounsaturated fatty acid side-chains (SFA/MUFA) in sphingolipids, glycerolipids (GL) and glycerophospholipids (GP) as well as reducing fat mass, circumferences and IR. Increased levels of polyunsaturated fatty acids in GPs and GLs were also observed, along with increased fat-free mass, VO_2_ max, and strength (all *P* < .05) after training.

**Conclusion:**

Our study revealed that 16 weeks of moderate-intensity CT remodeled the lipid metabolism in OB, and T2D individuals, even without dietary intervention, establishing a link between exercise-modulated lipid markers and mechanisms that reduce IR and obesity-related comorbidities.

Obesity and type 2 diabetes (T2D) are complex conditions with multifactorial influences, with genetics, environment, and lifestyle contributing to their pathogenesis (1). In recent years, there has been a considerable focus on research to comprehend the intricate molecular mechanisms underpinning these diseases. An increasing body of evidence indicates that alterations in lipid metabolism are pivotal in the genesis and progression of obesity and T2D (2, 3). Therefore, lipidomics may be important in discovering new mechanisms and lipid biomarkers linked with these metabolic disorders (4).

Lipids play vital roles in cellular membranes, acting as energy reserves, providing structural support, facilitating hormone synthesis, transporting vitamins, and enabling cell signaling, among other functions. Nonetheless, lipid metabolism dysregulation may result in obesity, where an excessive accumulation of body fat upsets the balance between energy intake and expenditure and contributes to ectopic deposition of lipids in the liver, skeletal muscle, and kidneys. White adipose tissue (WAT), the primary lipid storage location, experiences hypertrophy and hyperplasia in obesity, leading to elevated production and release of free fatty acids (FFA) into the bloodstream. This promotes insulin resistance (IR), a characteristic of T2D, as it impairs insulin signaling in peripheral tissues including skeletal muscle and liver (5, 6).

In addition, obesity is associated with changes in the composition of circulating lipid species, particularly increased concentrations of diacylglycerols (DG) and ceramides (Cer). These lipid species have been associated with the development of IR and β-cell dysfunction, both significant characteristics of T2D. Notably, DG have been demonstrated to trigger various protein kinase C isoforms, which disrupt insulin-signaling pathways. On the other hand, Cer are able to induce endoplasmic reticulum (ER) stress and apoptosis in pancreatic β-cells, resulting in impaired insulin secretion (7, 8).

Both quantitative and compositional changes have been observed in lipid species during obesity and T2D. Saturated fatty acids (SFAs) and monounsaturated fatty acids (MUFAs) are related to increased adiposity and IR (9, 10). In contrast, polyunsaturated fatty acids (PUFAs), in particular omega-3 fatty acids, have beneficial effects on health by reducing inflammation, improving insulin sensitivity, and preserving the function of pancreatic β cells (9). Moreover, dysregulation of desaturase enzymes associated with the production of specific lipid species, such as stearoyl-CoA desaturase-1 (SCD-1), has been linked to IR associated with obesity (10).

Regular physical exercise is an effective nonpharmacological therapy for IR, obesity, and related comorbidities (11) The most highly recommended intervention is combined training (CT), which merges the physiological benefits of aerobic training (AT) and resistance training (RT) (12-14). CT has demonstrated several favorable effects on body composition, comprising decreased fat mass (FM) and increased fat-free mass (FFM), leading to a reduction in waist circumference (WC) and decreased inflammation. Moreover, RT leads to improved cardiorespiratory fitness (maximum oxygen consumption: VO_2_max), increased strength, improved insulin sensitivity, and increased glucose uptake by muscles. It facilitates the mobilization of both visceral and subcutaneous fat, ultimately reducing IR in individuals with obesity and T2D (14, 15).

Our prior research has shown that CT has a favorable effect on the metabolome of middle-aged men who are obese, as well as adolescents who are overweight or obese (16-18). Certain serum metabolites were linked to improvements in VO_2_ max, decreases in FM, and improvements in IR. However, it remains uncertain whether regular exercise has the capacity to modify lipid species levels, both in plasma and WAT, and consequently affect the health of individuals facing obesity and associated comorbidities. Using a lipidomics approach, our aim was to investigate whether regular moderate-intensity exercise induces changes in plasma and WAT levels of lipid species in middle-aged individuals with normal weight, obesity, and T2D after 16 weeks of CT using an untargeted lipidomics approach based on ultra-high performance liquid chromatography-mass spectrometry (UHPLC-MS) to identify the major changes in lipid profile across classes. By correlating these changes with clinical markers, we sought to elucidate the potential mechanisms that underpin the ability of exercise to mitigate adverse outcomes associated with obesity and T2D.

## Material and Methods

All the procedures were approved by the research ethics committee of the Faculty of Medical Sciences of UNICAMP (protocol No. 1.597.626). This study was registered in the Brazilian Clinical Trials Registry (RBR-62n5qn), where the full trial can be accessed. All volunteers signed an informed consent form before starting the study. The procedures described in this article were conducted in accordance with the Helsinki Declaration (19).

### Participants

This study conducted a within-participant longitudinal trial in middle-aged volunteers of both sexes, aged between 40 and 60 years, divided into 3 groups with normal weight (NW, 52.2 ± 5.9 years), obesity (OB, 51.2 ± 5.5 years), and T2D (51.1 ± 3.9 years). Of the initial 491 individuals screened, 324 were excluded from the study during the initial interview (anamnesis) for not meeting the specified inclusion criteria. The study's inclusion criteria specified a sedentary lifestyle, meaning that all groups practiced less than 150 minutes of physical activity per week. The T2D group was to be diagnosed by a physician on the basis of a fasting plasma glucose greater than or equal to 126 mg/dL, and/or a glycemia 2 hours after an overload of 75 g of glucose 200 mg/dL or greater, and/or an glycated hemoglobin A_1c_ of 6.5% or greater. These criteria are based on the guidelines set by the Brazilian Diabetes Society. In addition, the T2D group was to maintain the same medication throughout the study, adjusting dosages only if medically advised. Individuals in the NW and OB groups were required to have a fasting blood glucose of less than 100 mg/dL and not have prediabetes. The body mass index (BMI) range selected for NW group was 18 to 27. This was based on a study showing that, due to the increase in obesity in the world, a BMI of up to 27 can now be considered a healthy weight with a low risk of death from all causes (20). For the OB and T2D group, the range was set at 27 to 35 to include individuals living with overweight and OB, with and without T2D. Individuals with diagnosed coronary artery disease, severe hypertension, on insulin therapy, thyroid disorders, using cigarettes, chronic obstructive pulmonary disease, liver/kidney diseases, limiting osteoarticular disease, or the use of medications, such as thiazolidinediones, β-blockers, anticoagulants, or anti-inflammatories, were excluded. Participants were instructed to maintain the same dosage of any other medication they were already taking, such as lipid-lowering drugs, hypertensive drugs, thyroid hormones, or vitamins, throughout the study.

**Figure 1.**
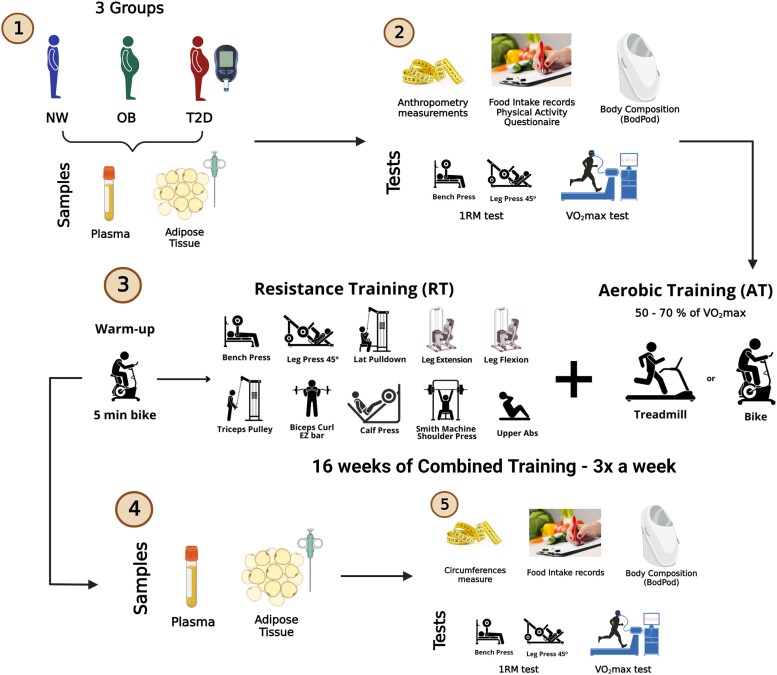
Representation of the experimental design. 1, Sample collection at the baseline. NW: normal weight, OB: obesity, and T2D: type 2 diabetes groups. 2, Assessments were performed at baseline (before starting the training program). 3, Combined training protocol intervention. 4, Sample collection after the training period. 5, Assessments performed after the training program. During the control period, the same procedures were performed, except for 16 weeks of training, during which time the volunteers remained sedentary. Created using BioRender.com.

To prevent an imbalance that would compromise statistical power, some volunteers were exclusively assigned to the training group due to dropouts during the project. At the end of the study, which included those who successfully completed the control and training interventions, the NW group consisted of 18 controls and 18 training individuals. The control group of the OB group comprised 29 volunteers, whereas the training group comprised 28 participants. Similarly, the T2D group had 17 volunteers in the control group and 21 participants in the training group. Further details on the recruitment process are displayed in Supplementary Fig. S1 (21), together with comprehensive information about the participants and the experimental methodology. Fig. 1 shows the experimental design and provides a visual representation of the assessments described next.

### Assessment of Physical Activity

To assess the usual physical activity levels of the volunteers, 2 questionnaires were used: Baecke's questionnaire, which surveyed habitual physical activities within the past 12 months, and the International Physical Activity Questionnaire (IPAQ), a validated abbreviated Brazilian version that concentrated on physical activity during the week leading up to the investigation (22, 23).

### Dietary Intake Monitoring

Food intake records (FRs) were used to assess dietary habits at the beginning and end of the control and training phases. Volunteers were provided with detailed guidelines and a manual to accurately describe their 24-hour dietary recalls. Participants were instructed to report their food and drink consumption on predetermined days, on 3 separate and nonconsecutive days (including at least 1 weekend day).

A trained nutritionist used DietPro Lite software to assess both caloric intake and the amounts and ratios of macronutrients (carbohydrates, lipids, and proteins). Food intake was determined by analyzing data from 3 dietary records. It is important to note that no nutritional interventions were made during the study, and participants were instructed to maintain a consistent dietary intake pattern throughout both the control and training phases.

### Anthropometry and Body Composition

Height and body weight (BW) were measured using a calibrated standard scale (Filizola), with a precision of 0.1 kg and 0.1 cm. BMI was calculated using the following formula: body weight (kg) × (height [m])^−^². Waist (WC), abdominal, and hip circumferences (cm) were measured using a tape measure, according to the anatomical references (perimeters). To ensure accuracy, these measurements were taken in triplicate, and the subsequent averages were computed. Comprehensive evaluation of body composition was achieved through the employment of whole-body plethysmography, using the BOD POD body composition system. The assessment followed both the guidelines stipulated in the equipment manual and previously established criteria (24).

### Muscle Strength and Cardiorespiratory Assessments

Muscular strength was evaluated by conducting a one-repetition maximum (1-RM) test on both bench press and leg press equipment (25). Cardiorespiratory fitness was measured on an Inbramed ATL model treadmill in Brazil, applying an incremental protocol analyzing breath by breath with K4b equipment from Cosmed (26). Additional details regarding these assessments are provided in the supplementary material (21).

### Chronic Combined Training Protocol

CT protocol incorporated both resistance training (RT) and aerobic training (AT) sessions that were conducted thrice weekly Mondays, Wednesdays, and Fridays over a 16-week period. The program was split into 2 stages. In the initial stage (S1), participants performed RT consisting of 10 exercises: leg press, leg extension, leg curls, bench press, lat pulldown, biceps curl using an EZ bar, triceps pulley, smith machine shoulder press, calf press, and upper abdominal crunches. The exercise routine consisted of 3 sets of 12 submaximal repetitions, followed by 1 minute of rest between sets. Next, a 35-minute AT session was completed on a treadmill or cycle ergometer at varying intensities: 3.5 minutes at 45% to 50% of VO_2_max, 14 minutes at 50% to 55% of VO_2_max, another 14 minutes at 55% to 65% of VO_2_max, and a final 3.5 minutes at 45% to 50% of VO_2_max (15).

In stage 2 (S2) of the CT, the RT session mirrored the S1 exercises and sets, but with 10 submaximal repetitions and 1 minute rest. While the AT regimen structure remained similar, the intensity zones were revised following a new VO_2_max test after week 8. This phase consisted of 3 and a half minutes at 50% to 55% of VO_2_max, 14 minutes at 55% to 65% of VO_2_max, another 14 minutes at 65% to 70% of VO_2_max, and finally, a further 3 and a half minutes at 50% to 55% of VO_2_max, as detailed in (15).The AT intensity was carefully monitored through corresponding heart rate measurements, consistently ranging between 40% and 70% of VO_2_max. Each session started with a 5-minute warmup on a cycle ergometer. Adjustments, primarily in RT loads, were made every 2 weeks to ensure loads remained within the 50% to 75% 1RM range. This facilitated a gradual and progressive training overload increase throughout the program.

### Blood Collection, White Adipose Tissue Sampling, and Biochemical Analysis

Blood was collected in Vacutainer brand tubes (Becton Dickinson Ltd), with an anticoagulant of EDTA, where 8 mL was collected prior to the start of the intervention, at the end of the 16-week control period, and within a window of 48 to 72 hours after the last CT session. All collections were carried out after a fasting period of 10 to 12 hours. They were conducted at the same time always, between 7 and 10 Am, following a period of abstaining from exercise and alcohol consumption for over 24 hours. The tubes were immediately centrifuged at 956*g* for 10 minutes at 4 °C. Following this, plasma samples were stored in a freezer set at −80 °C, awaiting further analysis.

WAT was sampled under local anesthesia from the subcutaneous fat on the right side of the waist, specifically at the iliac crest, in amounts of approximately 50 to 100 mg using a Bergstrom needle. The tissue fragments were stored in 2-mL microtubes with anti-RNAse protection and rapidly frozen using liquid nitrogen.

Fasting glucose levels were measured using an enzymatic method (25), and insulin levels were assessed through the chemiluminescence immunoassay technique, employing automated equipment. The quantification of total cholesterol, triglycerides (TG), high-density lipoprotein (HDL), and low-density lipoprotein (LDL) levels was carried out using commercially available kits from Roche Diagnostics GmbH (26). The Homeostasis Model Assessment (HOMA-IR) was used to estimate IR, according to the following formula: To calculate insulin resistance, we used [(fasting insulin (μU.mL-1) × fasting glucose (mg.dL-1)) × 22.5-1] (16, 27).

### Untargeted Lipidomics Method

#### Chemicals and reagents

LC-MS grade water, methanol (MeOH), isopropanol (IPA), hypergrade LC-MS acetonitrile (ACN), chloroform, ammonium formate, and ammonium acetate were purchased from Sigma-Aldrich (Merck KGaA). The internal standard (IS) (Splash Lipidomix Mass Spec Standard) was purchased from Avanti Polar Lipids.

#### Lipids liquid-liquid extraction for plasma

Lipid extraction from plasma was performed using a modified Folch method (27). To do so, we combined 20 µL of plasma samples with 600 mL of chloroform/methanol (2:1) within a glass vial. The mixture was vortexed for 10 seconds before 400 µL of chloroform/water (1:1) was added. After centrifuging at 4 °C and 4000 rpm (5810 R, Eppendorf) for 10 minutes, we transferred the organic phase (lower layer) carefully to a vial. This process was repeated for a second extraction. The samples were dried under a stream of nitrogen gas and stored in glass vials at −80 °C until analysis.

To reconstitute the samples, 100 µL of 1:1 methanol/chloroform was used and the mixture was vortexed to complete homogenization. Quality control (QC) samples were prepared by mixing 10 µL of each sample, 20 µL of IS (1:10), and 170 µL of IPA/ACN/water (2:1:1) to create a pool of samples. Serial dilutions of the QC were prepared at 75%, 50%, and 25% intervals to assess the linearity of individual lipid quantification. To prepare the standards, 10 µL of Splash Lipidomix and 90 µL of IPA/ACN/water (2:1:1) were added. The samples were prepared by adding 10 µL of reconstituted samples to a vial with insert, 10 µL IS (1:10) and 80 µL IPA/ACN/water (2:1:1).

#### White adipose tissue extraction

WAT extraction was adapted from Roberts et al (2014) (28), with modifications to minimize plastic usage. Approximately 20 mg of frozen WAT was combined with 400 µL of water: methanol (3:1) and mechanically disrupted using the TissueLyser (Qiagen) containing a stainless-steel metal ball inside the vial, for 15 minutes at 20 Hz. After the addition of chloroform/water (1.1 mL, 4:1), the mixture was transferred to a glass vial. After centrifugation for 10 minutes at 4 °C (5810 R, Eppendorf) at 4000 RPM, the organic phase was collected, and the procedure was repeated for a second extraction. The whole procedure was carried out over ice at 0 °C. Following this, the samples were dried and stored at −80 °C until analysis. The reconstitution of the samples consisted of 200 µL of methanol/chloroform (1:1), followed by vortexing to ensure homogeneity.

#### Ultra-high performance liquid chromatography–mass spectrometry method

Chromatography was conducted using an ultra-high performance LC (Dionex Ultimate 3000) equipped with an ACQUITY UPLC CSH C18, 130 Å, 1.7 µm, 2.1 mm × 150 mm column coupled to an Orbitrap Velos Pro MS (Thermo Scientific Fisher). The column temperature was set at 55 °C. The binary solvent for the positive mode was constituted by solvent A (ACN/water 60:40, 10 mM ammonium formate) and solvent B (IPA/ACN (90:10) and 10 mM ammonium formate). Ammonium acetate was used instead of ammonium formate when running in negative mode. The autosampler temperature was set at 4 °C.

The gradient was defined as 0 minutes, 40% B, 0.8 minutes, 43% B, 0.9 minutes, 50% B, 4.8 minutes, 54% B, 4.9 minutes, 70% B, 5.8 minutes, 81%, 8.0 minutes, 99% B, 8.5 minutes, 99% B, 8.6 minutes, 40% B and 10 minutes, 40% B. The eluent flow rate was set to 0.200 mL/min. Profile mode was used to collect MS data over a full scan range of m/z 100 to 2000 both in positive and negative ionization modes.

#### L**iquid chromatography–mass spectrometry** data processing

LC-MS data was processed using MS-DIAL 4.7 (29), which was set with the following parameters: soft ionization, type of chromatographic separation (conventional LC/MS or data-dependent LC-MS/MS), data profilers for MS1 and MS/MS, positive and negative ion modes, and lipidomic target omics. The supplementary material (21) contains the detailed MS-DIAL parameters.

The MS-DIAL data matrix was imported into Microsoft Excel for manual data filtering. WAT samples were normalized by weight. We applied a coefficient of variation filter to calculate (SD QCs/average QCs) × 100. The individual lipids in the QCs with a coefficient of variation greater than 20% were excluded, and the QCs’ serial dilution was examined. The features were filtered based on their linear behavior with the exclusion of features with *R*^2^ less than 0.8. Adducts, intensities, retention times, and noise/ratios were determined. Any missing values were replaced with the average of their corresponding nonmissing values, using K-nearest neighbor imputation, whereby the closest features (k = 10) were determined in terms of the Euclidean distance of the responses across all samples. To ensure comparability, normalization of lipid classes was achieved by either referencing the lipid splash or calculating the average of lipid species within each respective class.

### Statistical Analysis

Descriptive variables between the groups were compared through univariate analysis. We examined data distribution and homogeneity of variances by applying the Shapiro-Wilk and Levene tests. For assessing clinical marker alterations between training and control periods, we computed fold change values for each group: (post control/pre control) and (post training/pre training). Independent-samples *t* test was then used for variables with a normal distribution, while the Mann-Whitney test was used for variables with a nonnormal distribution. The data analyses were carried out using IBM SPSS Statistics for Windows, version 28.0. The data are presented as mean ± SD and the level of statistical significance was set at 5% (*P* < .05).

A multivariate statistical analysis was carried out using SIMCA software (version 17.0; Umetrics). A log transformation was applied to reduce variability among sample intensities, and Pareto scaling was employed to standardize the data. Orthogonal least squares discriminant analysis (OPLS-DA) was conducted to compare the control and training groups. The principal discriminative features were verified via s-plot graphs, using a threshold of *P* [1] and *P* (corr) [1] between −.05 and .05. Model validation and robustness were evaluated through permutation tests (100 permutations; *P* < .01) and cross-validation (*R*^2^ and Q^2^ values). Lipids identified by the s-plot were tested for significance using either a *t* test or a Mann-Whitney *U* test, followed by grouping based on species and number of double bonds. To correlate significant lipid changes with clinical markers, a Pearson correlation coefficient was used with correlation matrices for exploratory visualization. GraphPad Prism 10.0 software was used to generate graphical plots.

## Results

### Comparative Analysis of Descriptive Variables Post Training Across Groups

All groups demonstrated significant changes in body composition after completing the training program. There was a slight increase in FFM and a reduction in FM and circumference measurements in all groups (all *P* < .05). These effects were emphasized by large effect sizes, as indicated by Hedges’ g values in Table 1. Moreover, the OB and T2D groups exhibited a significant reduction in BW and BMI within the training groups compared to the control group (*P* < .05). Notably, VO_2_max showed a significant increase across all groups after the CT intervention (*P* < .05), along with gains in strength as represented by bench press and leg press 1RM test results (*P* < .05).

**Table 1.** Effects of combined training on body composition, physical fitness, and clinical markers in different population groups

Variables	CNW (n = 18)		TNW (n = 18)		*P*	Effect size (Hedges)	COB (n = 29)		TOB (n = 28)		*P*	Effect size (Hedges)	CT2D (n = 17)		TT2D (n = 21)		*P*	Effect size (Hedges)
	Mean	SD	Mean	SD			Mean	SD	Mean	SD			Mean	SD	Mean	SD		
**Body weight, kg**	1.004	0.024	1.002	0.024	.856	0.058	1.010	0.013	0.995	0.027	.**002**	**0**.**691**	1.016	0.019	0.993	0.019	**<.001**	**1**.**198**
**BMI**	1.004	0.024	1.002	0.024	.856	0.058	1.010	0.013	0.995	0.027	.**002**	**0**.**691**	1.016	0.019	0.993	0.019	**<.001**	**1**.**198**
**Insulin, mlU/L**	0.995	0.386	0.882	0.319	.129	0.311	1.137	0.414	0.926	0.370	.**014**	**0**.**532**	1.351	0.699	0.979	0.341	.089	**0**.**687**
**Glycemia, mg/dL**	1.042	0.082	1.013	0.070	.063	0.376	1.045	0.077	1.006	0.069	.**013**	**0**.**524**	1.182	0.201	0.865	0.133	**<.001**	**1**.**860**
**HOMA IR**	1.108	0.452	0.983	0.419	.142	0.281	1.208	0.501	0.936	0.410	.**002**	**0**.**586**	1.685	1.167	0.897	0.415	**.005**	**0**.**921**
**Triglycerides, mg/dL**	1.026	0.162	1.011	0.270	.913	0.069	1.062	0.318	0.954	0.376	.287	0.305	1.113	0.301	0.949	0.342	**.031**	**0**.**496**
**Cholesterol, mg/dL**	1.023	0.111	0.974	0.193	.523	0.309	1.065	0.153	0.941	0.176	.**010**	**0**.**736**	1.081	0.143	0.973	0.151	**.021**	**0**.**720**
**HDL, mg/dL**	1.032	0.094	1.041	0.139	.749	−0.074	1.021	0.109	1.048	0.322	.935	−0.113	0.965	0.098	1.012	0.094	.281	−0.483
**LDL, mg/dL**	1.031	0.147	1.006	0.173	.927	0.150	1.105	0.276	0.965	0.191	.**019**	**0**.**584**	1.093	0.187	0.999	0.191	.220	0.485
**Leg press, kg**	1.111	0.128	1.413	0.422	.**004**	**−0**.**947**	1.037	0.119	1.356	0.258	.**000**	**−1**.**558**	0.998	0.121	1.304	0.177	**<.001**	**−1**.**939**
**Bench press, kg**	1.134	0.237	1.393	0.279	.**001**	**−0**.**977**	1.018	0.065	1.347	0.251	.**000**	**−1**.**755**	0.988	0.115	1.293	0.220	**<.001**	**−1**.**654**
**VO_2_max, mL.kg^−1^.min^−1^**	0.999	0.140	1.126	0.083	.**004**	**−1**.**077**	1.029	0.087	1.107	0.111	.**000**	**−0**.**766**	1.027	0.081	1.150	0.107	**<.001**	**−1**.**248**
**WC, cm**	1.009	0.020	0.988	0.020	.**001**	**1**.**051**	1.012	0.018	0.977	0.029	.**000**	**1**.**396**	1.009	0.016	0.974	0.011	**<.001**	**2**.**430**
**Abdomen, cm**	1.004	0.020	0.971	0.028	.**000**	**1**.**347**	1.006	0.023	0.969	0.036	.**000**	**1**.**188**	1.013	0.013	0.973	0.017	**<.001**	**2**.**481**
**Hip, cm**	1.006	0.027	0.982	0.033	.**032**	**0**.**795**	1.004	0.020	0.984	0.032	.**005**	**0**.**749**	1.003	0.018	0.987	0.024	**.011**	**0**.**733**
**%FFM**	1.006	0.026	1.028	0.039	.**012**	**−0**.**665**	1.004	0.027	1.030	0.023	.**000**	**−1**.**031**	0.987	0.027	1.031	0.039	**<.001**	**−1**.**258**
**FFM, kg**	1.004	0.019	1.032	0.036	.**002**	**−0**.**964**	1.006	0.024	1.027	0.028	.**004**	**−0**.**800**	0.996	0.029	1.030	0.016	**<.001**	**−1**.**496**
**%FM**	1.007	0.079	0.931	0.066	.**002**	**1**.**007**	0.994	0.038	0.960	0.037	.**000**	**0**.**874**	1.031	0.068	0.957	0.033	**<.001**	**1**.**412**
**FM, kg**	1.001	0.079	0.926	0.071	.**003**	**0**.**973**	0.999	0.039	0.956	0.051	.**001**	**0**.**921**	1.044	0.075	0.957	0.035	**<.001**	**1**.**510**

Values are presented in mean (fold change post/pre) and SD. Effect sizes (Hedges) indicate the magnitude and direction of changes. Group differences are indicated by significant *P* values and large effect sizes in bold.

Abbreviations: BMI, body mass index; CNW, control normal weight; COB, control obese, CT2D, control type 2 diabetes; FFM, fat-free mass; FM, fat mass; HDL, high-density lipoprotein; HOMA IR, Homeostatic Model Assessment for Insulin Resistance; LDL, low-density lipoprotein; TNW, trained normal weight; TOB, trained obese; TT2D, trained type 2 diabetes; VO_2_max, maximum oxygen consumption; WC, waist circumference.

One of the findings was that the NW group did not exhibit any changes in biochemical markers after training. In contrast, obese patients had a significant reduction in glucose, insulin, cholesterol, and LDL levels in the OB (*P* < .05 and effect size > 0.5). The group with T2D showed a statistically significant reduction in plasma glucose and HOMA-IR variables, with a large effect size (see Table 1). Furthermore, alterations in TG and cholesterol were observed, indicating a partial reduction in dyslipidemia. However, no alterations were noted in the LDL and HDL cholesterol fractions.

The group diagnosed with T2D had an average duration of 5.5 ± 2.5 years of diabetes. After training, they showed a significant reduction in glycated hemoglobin A_1c_, with a variation of 0.91 and *P* = .02 (pre 7.73 ± 2.56 and post 7.05 ± 1.52), in addition to reductions in HOMA-IR and fasting glycemia already mentioned. Additionally, the volunteers remained on the same antiglycemic medication throughout the study, which included metformin, sulfonylurea, SGLT2 blockers, and DDP-4 inhibitors.

### Baseline Physical Activity Levels

The analysis of physical activity questionnaires revealed that volunteers were insufficiently active prior to participation in the project, which confirmed that they were sedentary. The NW group had an average of 115.5 ± 56.3 minutes of physical activity per week, while the OB group reported only 86.7 ± 50.8 minutes, and the T2D group had a total of 105.5 ± 66.0 minutes. No significant differences were detected among the groups.

### Dietary Assessment

The analysis of food records showed that there were no significant differences in the distribution of macronutrients (carbohydrates, proteins, and lipids) and total caloric intake between the precontrol, postcontrol, and posttraining groups (Supplementary Fig. S2) (21). However, the T2D group exhibited higher total calorie intake and carbohydrate consumption (Supplementary Fig. S2) (21) in comparison to the others. Interestingly, no differences were observed between the OB and NW groups.

### Lipidomic Alterations Induced by Exercise

**Figure 2.**
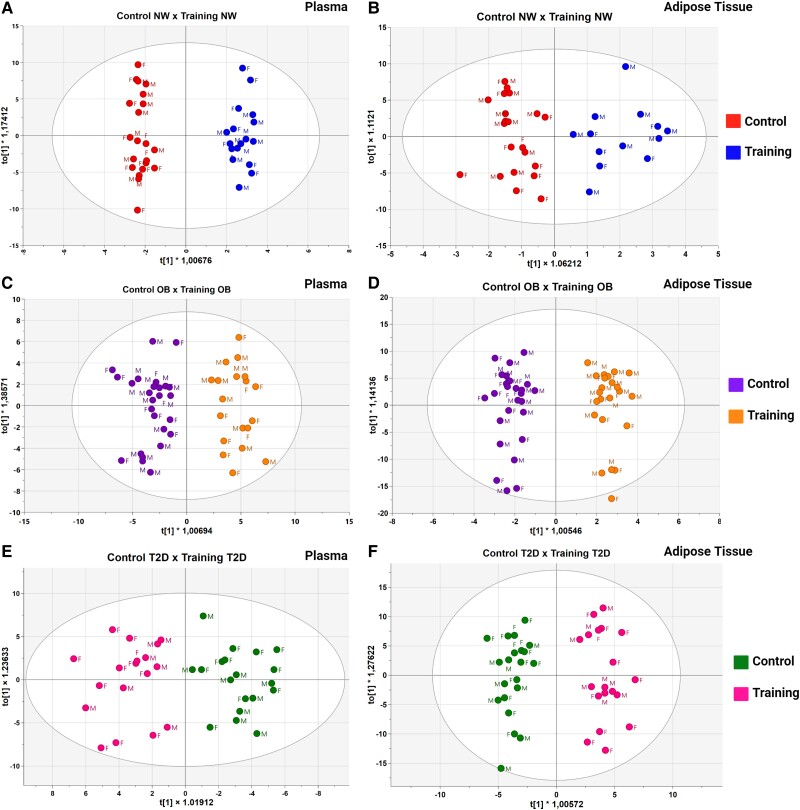
Comparison between control and training groups among individuals with normal weight (NW), obesity (OB), and type 2 diabetes (T2D) using orthogonal partial least squares discriminant analysis (OPLS-DA). Panels A, C, and E depict plasma samples obtained in positive ionization mode from NW, OB, and T2D groups, respectively. Panels B, D, and F show adipose tissue samples obtained in positive ionization mode. The labels “f” and “m” correspond to female and male samples. All models were validated with *Q*^2^ greater than or equal to 0.5 and the permutation tests *P* less than or equal to .05.

A total of 635 lipids were identified in plasma samples (444 in positive, and 191 in negative ionization mode). WAT samples consisted of 646 lipids (427 in positive and 219 in negative ionization mode). There was a clear separation between the control and training groups within the OPLS-DA displayed by all groups. The models for plasma and WAT, for the positive ionization mode of all groups, are presented in Fig. 2. The negative ionization mode models are available in the supplementary material (Supplementary Fig. S3) (21). All models were validated (Q^2^ ≥ 0.5 and permutation tests *P* < .05).

**Figure 3.**
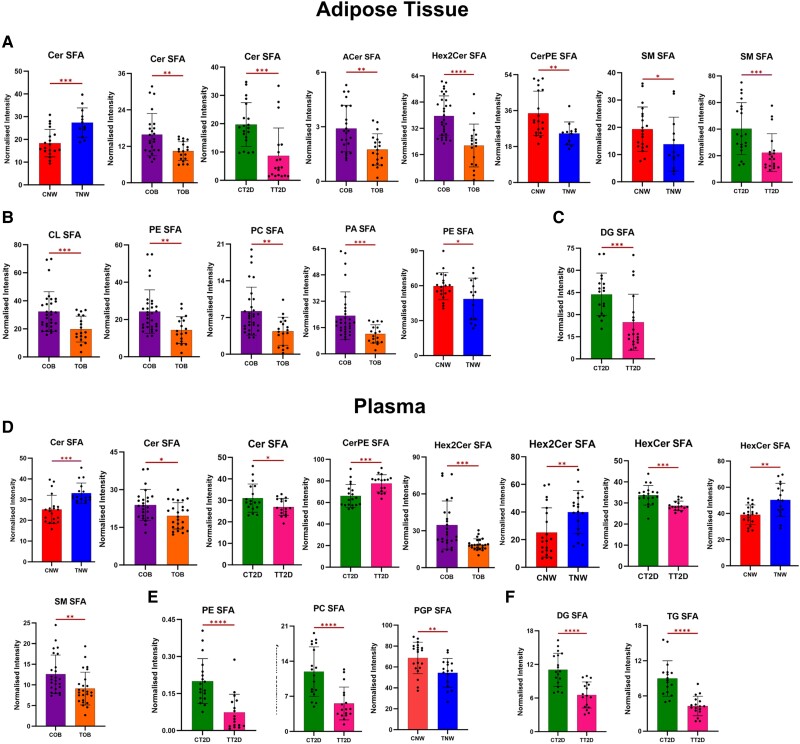
Alterations caused by exercise in lipid species containing long and very long chains of saturated fatty acids (SFA), in plasma and adipose tissue of individuals with normal weight (NW), obese (OB), and type 2 diabetes (T2D). The letter “C” before the name of the groups indicates control (CNW, COB, CT2D), and “T” indicates training (TNW, TOB, TT2D). A, B, and C—adipose tissue samples. D, E, F—plasma samples. The lipids were grouped into classes. A and D—sphingolipids (Ceramides [Cer], acylceramide [ACer], dihexosylceramide [Hex2Cer], ceramide phosphoethanolamine [CerPE], sphingomyelin [SM], hexosylceramide [HexCer]). B and E—glycerophospholipids (cardiolipin [CL], phosphoethanolamine [PE], phosphatidylcholine [PC], phosphatidic acid [PA], phosphatidylglycerol phosphate [PGP]). C and F—glycerolipids (diacylglycerol [DG], TG). Statistical significance is indicated by *P less than or equal to .05; **P less than or equal to .01; ***P less than or equal to .001; ****P less than or equal to .0001.

**Figure 4.**
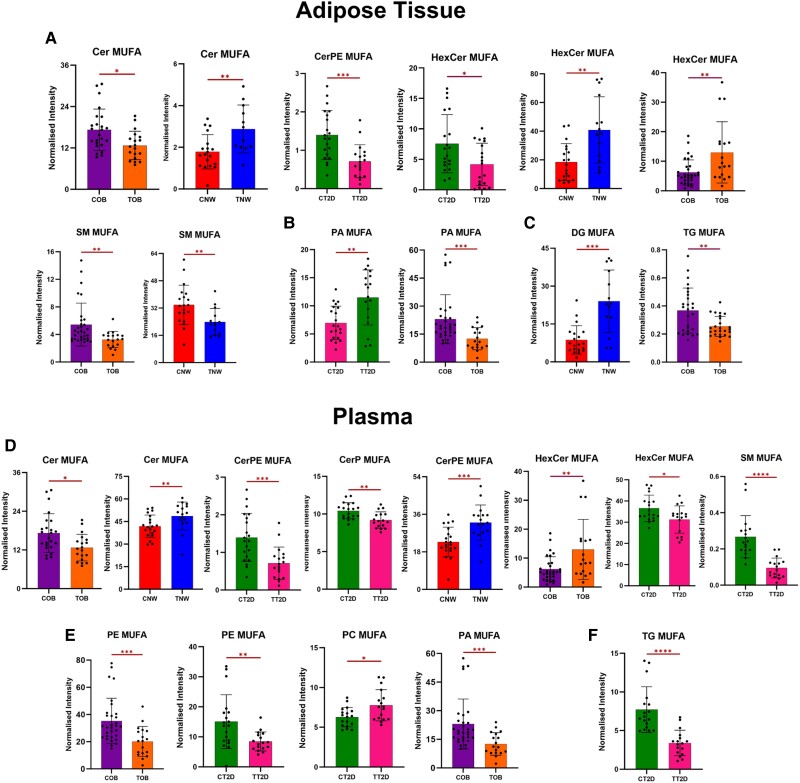
Alterations caused by exercise in lipid species containing long and very long chains of monounsaturated fatty acids (MUFA), in plasma and adipose tissue of individuals with normal weight (NW), obese (OB), and type 2 diabetes (TD2). The letter “C” before the name of the groups indicates control (CNW, COB, CT2D), and “T” indicates training (TNW, TOB, TT2D). A, B, and C—adipose tissue samples. D, E, F—plasma samples. A—sphingolipids (ceramides [Cer], ceramide phosphoethanolamine [CerPE], hexosylceramide [HexCer], sphingomyelin [SM]). B—glycerophospholipids (phosphatidic acid [PA]). C—Glycerolipids (diacylglycerol [DG], triacylglycerol [TG]). D—sphingolipids (ceramide phosphate [CerP]). E**—**glycerophospholipids (phosphoethanolamine [PE], phosphatidylcholine [PC], phosphatidic acid [PA]). F**—**glycerolipids [TG]). *P less than or equal to .05; **P less than or equal to .01; ***P less than or equal to .001; ****P less than or equal to .0001.

**Figure 5.**
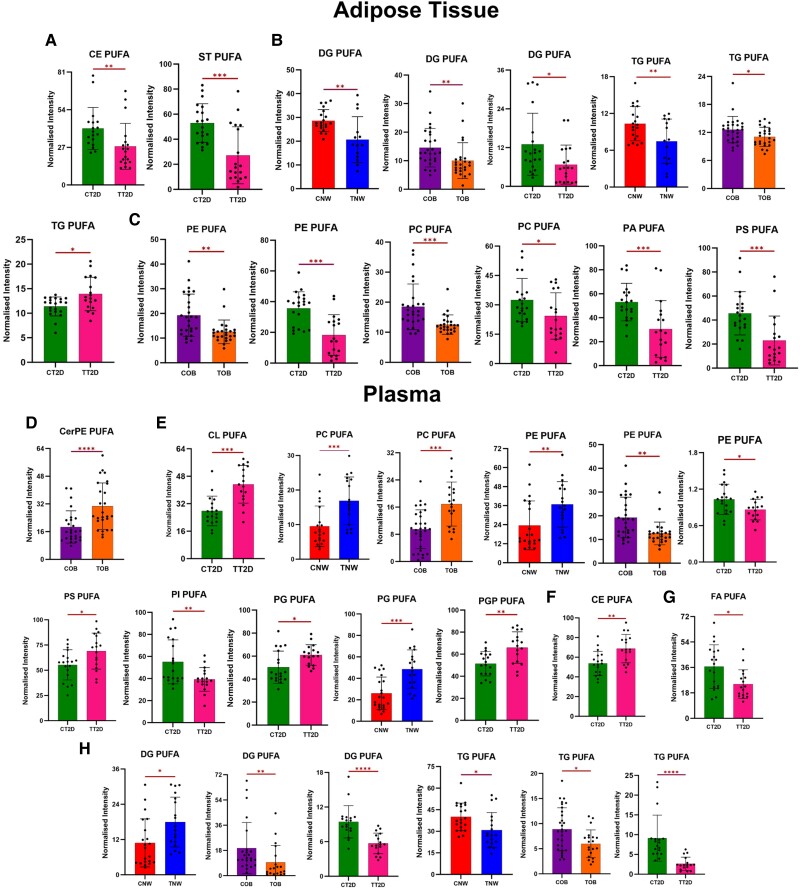
Main changes caused by exercise in lipid species containing long and very long chains of polyunsaturated fatty acids (PUFA), in plasma and adipose tissue of individuals with normal weight (NW), obese (OB), and type 2 diabetes (TD2). The letter “C” before the name of the groups indicates control (CNW, COB, CT2D), and “T” indicates training (TNW, TOB, TT2D). A, B, and C—adipose tissue samples. D, E, F—plasma samples. A—sterol lipids (cholesteryl ester [CE], sterol [ST]). B—Glycerolipids (diacylglycerol [DG], triacylglycerol [TG]). C—glycerophospholipids (phosphoethanolamine [PE], phosphatidylcholine [PC], phosphatidic acid [PA], phosphatidylserine [PS]). D**—**sphingolipids (ceramide phosphoethanolamine [CerPE]). E—glycerophospholipids (cardiolipin [CL], PE, PC, PA, phosphatidylinositol [PI], phosphatidylglycerol [PG], phosphatidylglycerol phosphate [PGP]). F—CE, G—fatty acid [FA], H—glycerolipids (DG, TG). *P less than or equal to .05; **P less than or equal to .01; ***P less than or equal to .001; ****P less than or equal to .0001.

**Figure 6.**
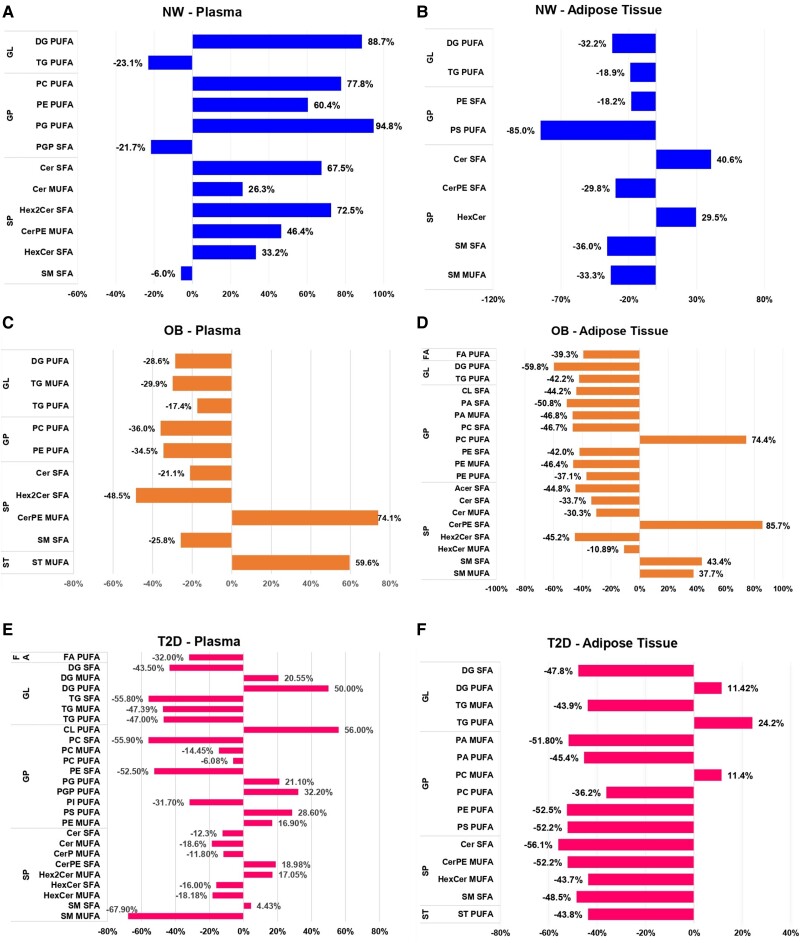
Percentage of variance in lipid species induced by 16 weeks of combined training in plasma samples from normal weight (NW), obese (OB), and type 2 diabetes (T2D) groups. A, C, and E represent plasma, and B, D, and F the adipose tissue.

Significant lipids were clustered from each comparison based on their specific species and the degree of saturation present within their fatty acid chains (SFA, MUFA, and PUFA) (Figs. 3-5). The selected lipids predominantly have long or very long chains. The detailed percentage variance after CT of each lipid species, divided by group and sample, is shown in Fig. 6, and all the lipids modified between groups are listed in Supplementary Tables S1, S2, and S3 in the supplementary material (21).

#### Impact of exercise on long- and very long-chain saturated fatty acid lipids

Statistically significant reductions (*P* < .05) were observed in the levels of long and very long-chain saturated fatty acid (VLSFA) chain lipids, both in the WAT and plasma of the 3 groups, but mainly in the WAT of obese individuals and in the plasma of T2D, as observed in Fig. 3. Among the sphingolipids (SP), there was a reduction of Cer, acylceramide (ACer), hexosylceramide (HexCer), dihexosylceramides (Hex2Cer), ceramide phosphoethanolamine (CerPE), and sphingomyelin (SM) (Fig. 3A—AT, and Fig. 3D—plasma). Glycerophospholipids (GP) exhibited alterations in WAT (Fig. 3B) and plasma (Fig. 3E) levels, including reductions in cardiolipins (CL), phosphatidylcholine (PC), phosphatidylethanolamine (PE), phosphatidic acid (PA), and phosphatidylglycerol phosphate (PGP). In the glycerolipid (GL) class, the training group showed lower levels of DG compared to the control, as observed in the plasma and WAT of participants with T2D, along with a decrease in plasma TG.

#### Metabolic changes in long- and very long-chain monounsaturated fatty acid side chains in plasma and white adipose t issue, induced by exercise

In contrast to the SFA lipid species, which predominantly exhibited reduced levels after 16 weeks of CT, the MUFA species exhibited a more diverse range of responses across groups and classes (Fig. 4). SP were the class with the highest number of altered species. The NW training group exhibited elevated levels of HexCer, and DG in WAT, along with increased concentrations of Cer and CerPE in plasma. On the contrary, the TOB (training OB) group showed decreased levels of Cer, SM, PA, and TG in WAT, along with increased levels of HexCer. In plasma, reduced levels of Cer, PE, and PA were observed. Notably, the TT2D (training T2D) group exhibited significant reductions in CerPE, HexCer, and TG levels in WAT, followed by an increase in PA levels. In plasma, reductions in ceramide phosphate (CerP), HexCer, SM, TG, and PE levels were observed, concomitant with an increase in plasma PC levels (all exhibiting significance of at least *P* < .05).

#### Metabolic changes in long- and very-long chain polyunsaturated fatty acids in plasma and white adipose tissue, induced by exercise

The classes that exhibited the highest number of altered PUFA lipid species were GPs and GLs (see Fig. 5). Within the NW training group, a decrease in TG and DG levels was observed in AT, while an increase in PE, PC, PG, and DG levels occurred in plasma samples. In the TOB group, WAT analysis revealed reduced levels of DG, TG, PE, and PC compared to COB. In plasma samples, an increase in CerPE and PC levels was observed, along with a decrease in PE, DG, and TG. Among the groups, T2D presented the most substantial alterations, including reductions in cholesterol ester (CE), sterol (ST), DG, PE, PC, PA, and phosphatidylserine (PS) levels in AT. In addition, there was an increase in TG levels. In the plasma, a decrease in PE, phosphatidylinositol (PI), FA, DG, and TG was observed, in addition to an increase in PS, phosphatidylglycerol (PG), phosphatidylglycerol phosphate (PGP), and CE (see Fig. 5—all exhibiting significance of at least *P* < .05). The results demonstrated that in WAT all GP PUFA showed reduced intensities after training, but in plasma, the majority showed increased levels, except PE (only the NW group increased) and PI.

#### Correlations between clinical markers and exercise-induced alterations in lipid species

We conducted a Pearson correlation analysis to investigate possible interrelationships between exercise-induced changes in lipid species and corresponding changes in clinical markers. We aimed to shed light on the underlying physiological mechanisms that connect lipid metabolism with clinical outcomes after CT intervention. All correlation matrix plots can be found in Supplementary Fig. S4 (21) and a summary with the main correlations are represented in Table 2.

**Table 2.** Correlation table summary for exercise-induced changes in lipid species and clinical markers across different groups and sample types

Group	Plasma			Adipose tissue		
	correlation comparison	*R* (Pearson)	*P*	correlation comparison	*R* (Pearson)	*P*
**NW**				**Cer SFA × VO_2_max**	−0.5704	≤.05
				**Cer SFA × %FFM**	−0.5376	≤.05
				**Cer SFA × %FM**	0.6063	≤.03
				**PE SFA × WC**	0.5252	≤.05
				**SM SFA × WC**	−0.6797	.01
				**TG PUFA × abdomen**	0.5492	.04
**OB**	**CerPE PUFA × VO_2_max**	−0.4634	.001	**Cer SFA × insulin**	−0.5534	.01
	**TG PUFA × WC**	−0.48283	.001	**Cer SFA × HOMA B**	−0.5076	.03
	**TG PUFA × insulin**	−0.4974	.001	**CerPE SFA × LDL**	0.4937	.03
	**TG PUFA × HOMA IR**	−0.5262	<.001	**PE PUFA × insulin**	−0.5176	.02
	**TG PUFA × HDL**	0.48	.001	**PE PUFA × HOMA IR**	−0.5171	.02
**T2D**	**CE PUFA × %FFM**	−0.567	.02	**CE PUFA × BMI**	0.58	.01
	**CE PUFA × %FM**	0.559	.02	**CE PUFA × WC**	0.511	.03
	**CE PUFA × FM (kg)**	0.5	.04	**CE PUFA × abdomen**	0.472	.04
	**CerPE MUFA × Cholesterol**	−0.595	.01	**Cer SFA × BMI**	0.62	<.001
	**CerPE MUFA × %FM**	−0.586	.01	**DG SFA × BMI**	0.54	.01
	**CerPE MUFA × VO_2_max**	0.492	≤.05	**CerPE MUFA × VO_2_max**	0.563	.01
	**CerPE MUFA × %FFM**	0.564	.02	**CerPE MUFA × %FM**	−0.475	.04
	**Cer SFA × %FFM**	−0.581	.01	**PA PUFA × BMI**	0.611	<.001
	**Cer SFA × %FM**	0.573	.02	**PA PUFA × abdomen**	0.498	.04
	**Cer SFA × FM (kg)**	0.649	<.001	**PC PUFA × Cholesterol**	0.481	.04
	**CerP MUFA × VO_2_max**	−0.498	.04	**PC PUFA × LDL**	0.532	.02
	**CerP MUFA × cholesterol**	0.506	.04	**PC PUFA × Cholesterol**	−0.666	<.001
	**DG PUFA × FM (kg)**	−0.536	.03	**PS PUFA × insulin**	−0.599	<.01
	**PC SFA × BMI**	0.497	.04	**PS PUFA × HOMA IR**	−0.602	<.01
	**PC SFA × insulin**	0.54	.03	**PS PUFA × TG**	−0.638	.004
	**PC SFA × HOMA B**	0.538	.03	**PS PUFA × waist**	−0.602	<.01
	**PGP PUFA × %FFM**	−0.57	.02	**ST PUFA × BMI**	0.595	<.01
	**PGP PUFA × %FM**	0.574	.01			
	**PGP PUFA × FM (kg)**	0.515	.04			
	**TG SFA × WC**	−0.628	<.01			
	**TG SFA × abdomen**	−0.531	.03			

Abbreviations: %FFM, fat free mass percentage; %FM, fat mass percentage; BMI, body mass index; CE, cholesterol ester; Cer, ceramide; CerPE, ceramide phosphatidylethanolamine; HDL, high-density lipoprotein; HOMA IR, Homeostatic Insulin Resistance Assessment Model; HexCer, hexosylceramide; LDL, low-density lipoprotein; MUFA, monounsaturated fatty acids; NW, normal-weight group; OB, obesity group; PA, phosphatidic acid; PC, phosphatidylcholine; PE, phosphatidylethanolamine; PGP, phosphatidylglycerol phosphate; PS, phosphatidylserine; PUFA, polyunsaturated fatty acids; SFA, saturated fatty acid; SM, sphingomyelin; ST, sterol lipids; T2D, type 2 diabetes group; TG, triacyclglycerols; VO_2_max, maximum oxygen consumption; WC, waist circumference. Pearson correlation coefficient: R.

Our findings exhibited associations between different types of lipids and markers related to biochemistry and body composition. Specifically, SFAs positively correlated with insulin levels, HOMA IR, cholesterol, LDL, BMI, WC, abdominal measurements, percentage of FM, and FM (kg). Conversely, these SFAs displayed negative correlations with percentage of FFM, HDL, and HOMA-B (β-cell function). It was also possible to note a negative correlation between Cer SFA and VO_2_max. On the other hand, lipids from MUFAs and PUFAs showed the opposite results, correlating positively with the benefits associated with physical training (FFM, VO_2_max, HDL, HOMA B) and negatively with the adverse effects linked to obesity and diabetes (BMI, HOMA-IR, cholesterol, LDL, circumferences, FM), as summarized in Table 2. Interestingly, certain MUFA lipids like PC, PA, and PGP displayed correlation patterns like SFA lipids rather than PUFAs. Additionally, CE PUFA and ST PUFA, both belonging to the sterol lipid class, showed positive correlations with BMI, abdominal measurements, and WC. PC PUFA correlated with VO_2_max and FFM, possibly being an interesting marker related to exercise results.

## Discussion

The aim of this study was to investigate the changes in lipidomic profiles of plasma and WAT of NW, OB, and T2D individuals after 16 weeks of moderate-intensity CT without dietary intervention. Additionally, alterations in body composition, physical fitness, and clinical markers after CT were assessed and associated with changes in lipid species. Not only did we observe reductions in long-chain saturated species (Cer, Acer, HexCer, Hex2Cer, SM, CerPE, CL, PE, PC, PA, DG, TG), but we also found positive correlations with percentage of FM, WC, abdomen, insulin, HOMA IR, and BMI; meanwhile, negative correlations were seen with percentage of FFM and VO_2_max. These results emphasize the powerful effects of exercise on enhancing overall health. Group differences were also observed in MUFA species (especially SP and GL) and PUFA (GP and GL). It is worth mentioning that this study is the first to use lipidomics to assess changes induced by a prescribed and controlled moderate-intensity exercise program in a middle-aged population of both sexes and with associated comorbidities. By highlighting the efficacy and key role of physical exercise in promoting the population's health, this research extends our understanding of how exercise can counteract the negative effects of noncommunicable diseases (NCDs) on lipid metabolism.

In terms of dietary monitoring, there were no significant differences in dietary patterns between the preintervention and postintervention/control periods, as reported by the participants. This outcome removes the possibility of confounders due to dietary changes during the study, increasing the reliability of the results. Interestingly, the T2D group exhibited a higher calorie and carbohydrate intake compared to the other groups. In contrast, the OB group showed no significant variances when compared to the NW group, despite their higher BW. From these observations, we hypothesized that the NW group likely has a higher proportion of percentage of FFM and potentially an increased basal metabolic rate, resulting in an increased daily energy expenditure. In contrast, the OB group may have exhibited reduced nonexercise thermogenesis when compared to the NW group, leading to a rise in FM. Finally, the OB group may have potentially underreported food intake during recalls, a commonly documented tendency in those living with OB (30). The clinical markers demonstrated that CT is a highly effective intervention for improving health outcomes in participants, even when they adhere to the minimum recommended amount and intensity of weekly exercise.

We noted a significant reduction in long and very long-chain Cer SFA and MUFA levels within the plasma and WATs of participants with obesity and T2D following the CT intervention. Higher levels of Cer have been associated with dysregulated insulin signaling through multiple mechanisms, including β-cell apoptosis, inflammatory responses, ER stress, and oxidative stress (31). Cer acts as an antagonist to insulin signaling, disrupting the signaling cascade by inhibiting phosphatidylinositol 3-kinase and blocking the activation of the anabolic signaling pathway Akt/PKB. This leads to Cer impairing glucose uptake, disrupting glycogen or TAG storage, activating protein phosphatase 2A, and triggering the release of proinflammatory cytokines. OB is closely associated with chronic inflammation resulting from an excess of FAs, which stimulates high levels of tumor necrosis factor-α and Toll-like receptors and activates Cer synthesis (32).

The literature has already shown that high plasma levels of Cer containing SFA are indicative of a high risk of T2D (33), and Cer can be very detrimental, especially in cardiovascular disease (CVD). For instance, specific species such as C24:0 and C24:1 have been associated with high mortality rates in chronic heart failure patients and increased all-cause mortality (34). The findings from our research provide further evidence of the significance of exercise in lowering Cer levels. We noticed a decline in plasma C24:0 levels among the OB group, as well as reduced C24:1 level in both the WAT of the OB group and the plasma of individuals with T2D (Supplementary Table S2 and S3 (21)). These results highlight the potential of CT as a protective measure against CVD.

Additionally, it has been previously reported that other SP species in the Cer family, such as SM, HexCer, Hex2Cer, CerPE, and CerP, have also been implicated in the risk of IR development, endothelial dysfunction, and atherosclerosis, which are closely associated with cardiometabolic diseases (35). The CerPE is structurally similar to SM aside from its headgroup, and demonstrated a negative correlation with cholesterol and FM. Despite sharing similarities with SM, CerPE has been poorly studied in the scientific literature and little attention has been paid to its biological functions. Nonetheless, our observations have uncovered a favorable correlation between CerPE MUFA, VO_2_max, and FFM, indicating a potential involvement of this marker in the biological mechanisms of physical exercise.

An intriguing finding of our research was that the NW group showed training-related changes that were different from those observed in the OB and T2D groups, particularly in relation to SP levels. Specifically, this was demonstrated by increased levels of VLSFA Cer both in AT and plasma, as well as high levels of Hex2Cer and HexCer in the plasma of the NW training group. Contrary to long-chain Cers (C16 or C18), which are associated with an increased risk of CV events, diabetes, and mortality, a recent review of literature has suggested that VLSFA lipids, including SPs, may be associated with a reduced risk of CVD, mortality, and healthy aging (36). VLSFAs are synthesized endogenously from C18:0 in the ER through the activity of elongases belonging to the ELOVL enzyme family. The exact equilibrium between dietary intake and metabolic processes in circulating VLSFA levels remains unclear. However, this study has observed that exercise can enhance elongase enzyme activity, and as this group is unlikely to have an established inflammatory process like the others, this increase in SP VLSFA can be considered beneficial.

Another lipid class that showed changes with training was GL, which represent a large portion of the total lipids found in human plasma, and the concentration of TGs can increase because of genetic determinants, obesity, mitochondrial dysfunction (37), uncontrolled diabetes mellitus, and a sedentary lifestyle, causing hypertriglyceridemia (dyslipidemia strongly associated with premature risk of coronary artery disease) (38). Although TGs themselves are not signaling molecules, there is some evidence to suggest that lipids generated through the synthesis and breakdown of TGs may contribute to the development of IR. These lipids can disrupt the intracellular insulin signaling pathway, and include FFAs, DGs, and Cers (39). High levels of FFAs in the blood induce IR, triggering the activation of serine/threonine kinases, which in turn decrease tyrosine phosphorylation on insulin receptor substrates 1 and 2 (IRS 1 and 2), disrupting the IRS/PI3 K pathway, leading to a decrease in GLUT4 translocation to the cell membrane, generating an IR state (5). Furthermore, FFAs contribute to the generation of reactive oxygen species (ROS), primarily through the activation of NADPH oxidase via protein kinase C. These events of ROS production contribute to oxidative stress (40).

A previous study showed a positive correlation between TGs (SFA and MUFA) and IR, highlighting the importance of examining FA chain length and its saturation level within TGs, rather than focusing solely on total TG concentration (41). TGs both in plasma and WAT, especially those containing SFA (in the T2D group) and MUFA (in the OB and T2D groups), were reduced after 16 weeks of CT. Regular exercise reduces plasma TG levels through multiple mechanisms, including enhancing the activity of lipoprotein lipase, an enzyme responsible for TG breakdown in circulating lipoproteins; facilitating the oxidation of FAs in muscle cells, which decreases the availability of TG for secretion into the bloodstream; improving insulin sensitivity, leading to reduced very-LDL secretion from the liver and lower plasma TG levels; and decreasing BMI and FM, which are associated with lower TG levels (42). Decreased levels of TG containing PUFAs were observed in the plasma of all training groups when compared to their respective control groups. Negative correlations between these lipids and insulin, HOMA-IR, and WC were also detected.

The T2D group showed increased levels of long-chain TG PUFA in WAT. Physical exercise has been shown to modify desaturase and elongase enzymes, leading to an increase in MUFA and PUFAs, and a reduction in SFA chains (43). In addition, excessive FA levels in the bloodstream, caused by high visceral lipolytic activity, can result in several adverse effects, such as lipotoxicity, increased ROS and IR (44). Consequently, we hypothesized that CT remodeled the AT by enhancing enzymatic activity, elongating FAs in TG molecules, and introducing double bonds. This process appears to contribute to the reduction of FA levels in the bloodstream, as illustrated in Fig. 5G, improving IR.

Another crucial factor in mitigating IR is the maintenance of membrane properties, with a specific emphasis on membrane fluidity. The fluidity of cell membranes is crucial in several cellular functions and can be affected by conditions such as OB and diabetes (45). These processes include insulin secretion by β cells, glucose transport, and metabolic rate regulation, among others. Notably, a poor membrane fluidity resulting from an excess of SFA in phospholipids impairs insulin receptor signaling, which is essential for facilitating the transport of GLUT4 to the plasma membranes of muscle cells and adipocytes, a critical step in glucose uptake from the bloodstream, leading to its accumulation (46). In addition, changes in the composition of GP membranes in adipocytes contribute to inflammation in individuals with OB (47).

Our study demonstrated that CT altered the GP composition of membranes, reducing the levels of SFA species and increasing some MUFA or PUFA side chains in GP in plasma and AT. This information corroborates studies that have already reported alterations in GPs with exercise in WAT of rats (48) and skeletal muscles of athletes’ vs untrained men (49). One study showed that an increase in plasma MUFA lipids with dietary changes can improve insulin sensitivity. It seems that even without changing your diet and just exercising, it is also possible to promote changes in cell membranes and improve IR and health, especially for OB and T2D individuals.

One noteworthy observation among the various changes in GPs is the increase in plasma PS PUFA in the T2D group. Previous research has indicated that individuals with diabetes commonly exhibit elevated levels of PS SFA and reduced levels of PUFA. Nonetheless, it is possible to increase these levels by improving insulin sensitivity and regulating insulin signaling. This increase in PS PUFA is also linked to an improvement in mitochondrial dysfunction associated with diabetes (50). A negative correlation was observed between PS PUFA and HOMA-IR, which supports these findings.

Another CT-induced change worth noting is the increase in plasma CL levels in the T2D group. Regular physical exercise induces several beneficial adaptations within mitochondria, including increased mitochondrial density, improved function, reduced oxidative stress and increased structural and functional integrity of CLs within the inner mitochondrial membrane (51). CLs plays a fundamental role in facilitating oxidative phosphorylation and the synthesis of adenosine triphosphate, contributing to the efficient functioning of enzymes and mitochondrial complexes involved in energy production (52). These molecules may be damaged by the production of ROS, and their remodeling has been linked to mitochondrial dysfunction in conditions such as OB, diabetes, heart failure, and aging (53). The T2D training group, like the other training groups, increased their VO_2_max, decreased IR, possibly decreased oxidative stress, increased CL levels, and promoted the improvement of mitochondrial dysfunction associated with diabetes.

**Figure 7.**
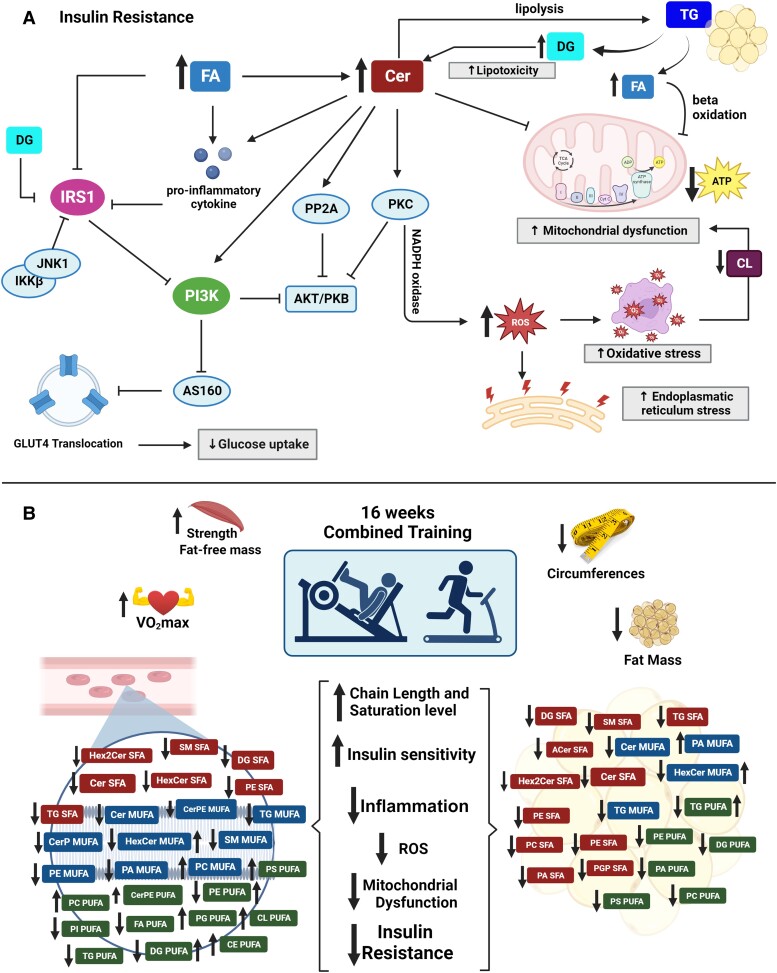
Mechanisms of insulin resistance and the relationship with the benefits of combined training on the lipidome of middle-aged individuals. A, An overview of the primary mechanisms that drive insulin resistance, focusing on the essential roles played by ceramides (Cer), fatty acids (FA), diacylglycerol (DG), triacylglycerol (TG), cardiolipins (CL) reactive oxygen species (ROS), adenosine triphosphate (ATP), insulin receptor substrate 1 (IRS1), phosphatidylinositol-3 kinase (PI3K), protein phosphatase 2A (PP2A), and protein kinase C (PKC). Arrows linking lipids and metabolic events represent leads to (→) or inhibits (ⱶ). Thicker arrows next to lipid or metabolic events represent an increase (↑) or decrease (↓), respectively. B, Combined training (CT)-induced changes in the lipid profile of plasma and adipose tissue of individuals, contributing to the reduction of insulin resistance (IR) and other associated health benefits. The lipid species were colored to facilitate visualization, with red being SFA: saturated fatty acid chain, blue being MUFA: monounsaturated fatty acid chain, and green PUFA: polyunsaturated fatty acid chain. VO_2_max (maximum oxygen consumption). Sphingolipids: ceramide (Cer), hexosylceramide (HexCer), dihexosylceramide (Hex2Cer), sphingomyelin (SM), phosphoethanolamine ceramide (CerPE), acylceramide (ACer). Glycerolipids: triacylglycerol (TG), diacylglycerol (DG). Fatty acids (FA). Glycerophospholipids: cardiolipins (CL), phosphatidylcholine (PC), phosphatidylethanolamine (PE), phosphatidic acid (PA), phosphatidylglycerol (PG), phosphatidylglycerol phosphate (PGP), phosphatidylserine (PS), phosphatidylinositol (PI). Sterol lipids: cholesterol ester (CE). Created using BioRender.com.

Our data highlight the multiple benefits of CT and provide valuable insights into the interplay between exercise, lipid metabolism, and insulin sensitivity. Fig. 7A presents a summary of the main mechanisms underlying IR and how some of the lipids we measured play a critical part in it. In Fig. 7B, we described the alterations induced by CT both in plasma and WAT that contributed to the reduction of IR and other benefits. The findings strongly indicate that exercise can effectively counteract the harmful effects associated with OB and T2D by modulating the levels of these lipids, thus promoting overall health improvements in all three groups.

It is important to consider the limitations of our study. First, we assessed IR using the HOMA-IR method, which, although widely used, may not provide the same level of accuracy as a hyperinsulinemic euglycemic clamp. Second, our dietary monitoring, although consistent throughout the study, was based on relatively short-term dietary records obtained at baseline and at the end of the study. There may have been changes in dietary composition over time that are not fully reflected in these records. The sample size of our participant pool was limited due to logistical reasons, extensive project evaluations, and the need for extensive monitoring and control of all training sessions to ensure the effectiveness of the training. In addition, we obtained a smaller number of biopsies compared to plasma samples because we experienced difficulties in collecting biopsies by our collaborating physicians, and some volunteers did not agree to have biopsies collected, only plasma. It is important to bear these limitations in mind when interpreting the results of our study. Finally, despite all the evidence pointing to exercise as the main driver of lipidomic changes, no additional experiments were performed in this study to prove this mechanistically and demonstrate that the lipid changes were caused by exercise, or the subsequent weight loss or body composition change toward more lean mass. This work should be considered as a starting point to guide future research.

## Conclusion

In this study, we have shown that just 16 weeks of combined moderate-intensity training can have a powerful effect on metabolic health, where training effectively reduced the levels of lipid species with SFA and MUFA chains, particularly within SP (Cer, HexCer, Hex2Cer, SM), GL (DG, TG), and GP (PC, PE, PA), which have strong associations with IR, inflammation, and mitochondrial dysfunction. The study identified positive correlations of these lipids with FM, BMI, HOMA-IR, circumferences, and LDL, and negative correlations with VO_2_max, FFM, and HOMA-B. In addition, CT also increased the length and saturation of PUFA side chains in TG, PC, PG, PS, and CL in the T2D group, showing a strong association with increased insulin sensitivity and decreased IR, reducing the risk of developing CVD. Essentially, our study highlighted how regular, moderate-intensity exercise can reshape our lipid metabolism and promote a healthier state, by reducing the metabolic damage caused by OB and T2D, even without dietary intervention.

## Data Availability

Original data generated and analyzed during this study are included in this published article or in the data repositories listed in “References.”

## Abbreviations

AT: aerobic training
BMI: body mass index
BW: body weight
Cer: ceramides
CerPE: ceramide phosphoethanolamine
CL: cardiolipin
CNW: control normal weight
COB: control obese
CT: combined training
CT2D: control type 2 diabetes
CVD: cardiovascular disease
DG: diacylglycerols
ER: endoplasmic reticulum
FFA: free fatty acids
FFM: fat-free mass
FM: fat mass
GL: glycerolipids
GP: glycerophospholipids
HDL: high-density lipoprotein
HOMA IR: Homeostatic Model Assessment for Insulin Resistance
IR: insulin resistance
IS: internal standard
LDL: low-density lipoprotein
MUFA: monounsaturated fatty acid side chains
NW: normal weight
OB: obesity
OPLS-DA: orthogonal least squares discriminant analysis
PUFA: polyunsaturated fatty acids
QC: quality control
ROS: reactive oxygen species
RT: resistance training
S1: stage 1
S2: stage 2
SFA: saturated fatty acid side chains
SP: sphingolipids
T2D: type 2 diabetes
TG: triglycerides
TNW: trained normal weight
TOB: trained obese
TT2D: trained type 2 diabetes
UHPLC-MS: ultra-high performance liquid chromatography–mass spectrometry
VLSFA: very long-chain saturated fatty acid
VO_2_max: maximum oxygen consumption
WAT: white adipose tissue
WC: waist circumference

## References

[1] Blüher M . Obesity: global epidemiology and pathogenesis. Nat Rev Endocrinol. 2019;15(5):288‐298. 30814686 10.1038/s41574-019-0176-8

[2] Koay YC , Coster ACF, Chen DL, et al Metabolomics and lipidomics signatures of insulin resistance and abdominal fat depots in people living with obesity. Metabolites. 2022;12(12):1272. 36557310 10.3390/metabo12121272 PMC9781703

[3] Savage DB , Petersen KF, Shulman GI. Disordered lipid metabolism and the pathogenesis of insulin resistance. Physiol Rev. 2007;87(2):507‐520. 17429039 10.1152/physrev.00024.2006 PMC2995548

[4] Abinaya B , Waseem M, Kashif M, Srinivasan H. Lipidomics: an excellent tool for chronic disease detection. Curr Res Transl Med. 2022;70(4):103346. 35487168 10.1016/j.retram.2022.103346

[5] Shetty S , Kumari S. Fatty acids and their role in type–2 diabetes (review). Exp Ther Med. 2021;22(1):706. 34007315 10.3892/etm.2021.10138 PMC8120551

[6] Arner P , Rydén M. Fatty acids, obesity and insulin resistance. Obes Facts. 2015;8(2):147‐155. 25895754 10.1159/000381224 PMC5644864

[7] Petersen MC , Shulman GI. Roles of diacylglycerols and ceramides in hepatic insulin resistance. Trends Pharmacol Sci. 2017;38(7):649‐665. 28551355 10.1016/j.tips.2017.04.004 PMC5499157

[8] Sokolowska E , Blachnio-Zabielska A. The role of ceramides in insulin resistance. Front Endocrinol (Lausanne). 2019;10:577. 31496996 10.3389/fendo.2019.00577 PMC6712072

[9] Tortosa-Caparrós E , Navas-Carrillo D, Marín F, Orenes-Piñero E. Anti-inflammatory effects of omega 3 and omega 6 polyunsaturated fatty acids in cardiovascular disease and metabolic syndrome. Crit Rev Food Sci Nutr. 2017;57(16):3421‐3429. 26745681 10.1080/10408398.2015.1126549

[10] Liu X , Strable MS, Ntambi JM. Stearoyl CoA desaturase 1: role in cellular inflammation and stress. Adv Nutr. 2011;2(1):15‐22. 22211186 10.3945/an.110.000125 PMC3042787

[11] Bull FC , Al-Ansari SS, Biddle S, et al World health organization 2020 guidelines on physical activity and sedentary behaviour. Br J Sports Med. 2020;54(24):1451‐1462. 33239350 10.1136/bjsports-2020-102955 PMC7719906

[12] Maiorana A , O’Driscoll G, Goodman C, Taylor R, Green D. Combined aerobic and resistance exercise improves glycemic control and fitness in type 2 diabetes. Diabetes Res Clin Pract. 2002;56(2):115‐123. 11891019 10.1016/s0168-8227(01)00368-0

[13] Libardi CA , De Souza GV, Cavaglieri CR, Madruga VA, Chacon-Mikahil MPT. Effect of resistance, endurance, and concurrent training on TNF-α, IL-6, and CRP. Med Sci Sports Exerc. 2012;44(1):50‐56. 21697747 10.1249/MSS.0b013e318229d2e9

[14] Bonfante ILP , Chacon-Mikahil MPT, Brunelli DT, et al Combined training, FNDC5/irisin levels and metabolic markers in obese men: a randomised controlled trial. Eur J Sport Sci. 2017;17(5):629‐637. 28287024 10.1080/17461391.2017.1296025

[15] Bonfante ILP , Monfort-Pires M, Duft RG, et al Combined training increases thermogenic fat activity in patients with overweight and type 2 diabetes. Int J Obes. 2022;46(6):1145‐1154. 10.1038/s41366-022-01086-3 35173278

[16] Duft RG , Castro A, Bonfante ILP, et al Altered metabolomic profiling of overweight and obese adolescents after combined training is associated with reduced insulin resistance. Sci Rep. 2020;10(1):16880. 33037261 10.1038/s41598-020-73943-y PMC7547065

[17] Duft RG , Castro A, Bonfante ILP, Brunelli DT, Chacon-Mikahil MPT, Cavaglieri CR. Metabolomics approach in the investigation of metabolic changes in obese men after 24 weeks of combined training. J Proteome Res. 2017;16(6):2151‐2159. 28492082 10.1021/acs.jproteome.6b00967

[18] Duft RG , Castro A, Bonfante ILP, et al Serum metabolites associated with increased insulin resistance and low cardiorespiratory fitness in overweight adolescents. Nutr MetabolCardiovasc Dis. 2022;32(1):269‐278. 10.1016/j.numecd.2021.09.024 34906412

[19] General Assembly of the World Medical Association . World medical association declaration of Helsinki: ethical principles for medical research involving human subjects. J Korean Med Assoc. 2014;57(11):899.

[20] Afzal S , Tybjærg-Hansen A, Jensen GB, Nordestgaard BG. Change in body mass index associated with lowest mortality in Denmark, 1976-2013. JAMA. 2016;315(18):1989‐1996. 27163987 10.1001/jama.2016.4666

[21] Duft RG , Bonfante ILP, Palma-Duran SA, Chacon-Mikahil MP, Griffin JL, Cavaglieri CR. Supplemental files from “Moderate-intensity Combined Training Induces Lipidomic Changes in Individuals With Obesity and Type 2 Diabetes”. *Figshare*. Acessed February 19, 2024. 10.6084/m9.figshare.25243594 PMC11318996 38488044

[22] Baecke J , Burema J, Frijters J. A short questionnaire for the measurement of habitual physical activity in epidemiological studies. Am J Clin Nutr. 1982;36(5):936‐942. 7137077 10.1093/ajcn/36.5.936

[23] Bauman A , Ainsworth BE, Sallis JF, et al The descriptive epidemiology of sitting. Am J Prev Med. 2011;41(2):228‐235. 21767731 10.1016/j.amepre.2011.05.003

[24] Fields DA , Higgins PB, Hunter GR. Assessment of body composition by air-displacement plethysmography: influence of body temperature and moisture. Dyn Med. 2004;3(1):3. 15059287 10.1186/1476-5918-3-3 PMC411054

[25] Brown LE , Weir JP. ASEP procedures recommendation I: accurate assessment of muscular strength and power. J Exerc Physiol Online. 2001;4(3):1‐21.

[26] Libardi CA , Souza GV, Gáspari AF, et al Effects of concurrent training on interleukin-6, tumour necrosis factor-alpha and C-reactive protein in middle-aged men. J Sports Sci. 2011;29(14):1573‐1581. 21933039 10.1080/02640414.2011.609896

[27] Matthews DR , Hosker JP, Rudenski a S, Naylor BA, Treacher DF, Turner RC. Homeostasis model assessment: insulin resistance and beta-cell function from fasting plasma glucose and insulin concentrations in man. Diabetologia. 1985;28(7):412‐419. 3899825 10.1007/BF00280883

[28] Roberts LD , West JA, Vidal-Puig A, Griffin JL. Methods for performing lipidomics in white adipose tissue. *Methods Enzymol.* 2014;538:211–231. 10.1016/B978-0-12-800280-3.00012-8 24529441

[29] Tsugawa H , Cajka T, Kind T, et al MS-DIAL: data-independent MS/MS deconvolution for comprehensive metabolome analysis. Nat Methods. 2015;12(6):523‐526. 25938372 10.1038/nmeth.3393 PMC4449330

[30] Wehling H , Lusher J. People with a body mass index ⩾30 under-report their dietary intake: a systematic review. J Health Psychol. 2019;24(14):2042‐2059. 28810493 10.1177/1359105317714318

[31] Mandal N , Grambergs R, Mondal K, Basu SK, Tahia F, Dagogo-Jack S. Role of ceramides in the pathogenesis of diabetes mellitus and its complications. J Diabetes Complications. 2021;35(2):107734. 33268241 10.1016/j.jdiacomp.2020.107734 PMC8663915

[32] Carrard J , Gallart-Ayala H, Weber N, et al How ceramides orchestrate cardiometabolic health—an ode to physically active living. Metabolites. 2021;11(10):675. 34677390 10.3390/metabo11100675 PMC8538837

[33] Fretts AM , Jensen PN, Hoofnagle AN, et al Plasma ceramides containing saturated fatty acids are associated with risk of type 2 diabetes. J Lipid Res. 2021;62:100119. 34555371 10.1016/j.jlr.2021.100119 PMC8517199

[34] Shu H , Peng Y, Hang W, Li N, Zhou N, Wang DW. Emerging roles of ceramide in cardiovascular diseases. Aging Dis. 2022;13(1):232‐245. 35111371 10.14336/AD.2021.0710 PMC8782558

[35] Chew WS , Torta F, Ji S, et al Large-scale lipidomics identifies associations between plasma sphingolipids and T2DM incidence. JCI Insight. 2019;4(13):e126925. 10.1172/jci.insight.126925 PMC6629294 31162145

[36] Lemaitre RN , King IB. Very long-chain saturated fatty acids and diabetes and cardiovascular disease. Curr Opin Lipidol. 2022;33(1):76‐82. 34907969 10.1097/MOL.0000000000000806 PMC8702474

[37] Prasun P . Mitochondrial dysfunction in metabolic syndrome. Biochim Biophys Acta (BBA)—Mol Basis Dis. 2020;1866(10):165838. 10.1016/j.bbadis.2020.165838 32428560

[38] Quehenberger O , Dennis EA. The human plasma lipidome. N Engl J Med. 2011;365(19):1812‐1823. 22070478 10.1056/NEJMra1104901 PMC3412394

[39] Al-Sulaiti H , Diboun I, Banu S, et al Triglyceride profiling in adipose tissues from obese insulin sensitive, insulin resistant and type 2 diabetes mellitus individuals. J Transl Med. 2018;16(1):175. 29940972 10.1186/s12967-018-1548-x PMC6019324

[40] Cruz NG , Sousa LP, Sousa MO, Pietrani NT, Fernandes AP, Gomes KB. The linkage between inflammation and type 2 diabetes mellitus. Diabetes Res Clin Pract. 2013;99(2):85‐92. 23245808 10.1016/j.diabres.2012.09.003

[41] Sanders F , McNally B, Griffin JL. Blood triacylglycerols: a lipidomic window on diet and disease. Biochem Soc Trans. 2016;44(2):638‐644. 27068982 10.1042/BST20150235

[42] Durstine JL , Grandjean PW, Cox CA, Thompson PD. Lipids, lipoproteins, and exercise. J Cardiopulm Rehabil. 2002;22(6):385‐398 12464825 10.1097/00008483-200211000-00002

[43] Nono Nankam PA , Mendham AE, van Jaarsveld PJ, et al Exercise training alters red blood cell fatty acid desaturase indices and adipose tissue fatty acid profile in African women with obesity. Obesity. 2020;28(8):1456‐1466. 32627952 10.1002/oby.22862

[44] Sobczak AIS , Blindauer CA, Stewart AJ. Changes in plasma free fatty acids associated with type-2 diabetes. Nutrients. 2019;11(9):2022. 31466350 10.3390/nu11092022 PMC6770316

[45] Ahyayauch H . Relationship between obesity, insulin resistance and cell membrane properties. Eur J Clin Exp Med. 2023;21(2):357‐364.

[46] Pilon M . Revisiting the membrane-centric view of diabetes. Lipids Health Dis. 2016;15(1):167. 27671740 10.1186/s12944-016-0342-0 PMC5037885

[47] Pietiläinen KH , Róg T, Seppänen-Laakso T, et al Association of lipidome remodeling in the adipocyte membrane with acquired obesity in humans. PLoS Biol. 2011;9(6):e1000623. 21666801 10.1371/journal.pbio.1000623 PMC3110175

[48] May FJ , Baer LA, Lehnig AC, et al Lipidomic adaptations in white and brown adipose tissue in response to exercise demonstrate molecular Species-specific remodeling. Cell Rep. 2017;18(6):1558‐1572. 28178530 10.1016/j.celrep.2017.01.038 PMC5558157

[49] Andersson A , Sjödin A, Hedman A, Olsson R, Vessby B. Fatty acid profile of skeletal muscle phospholipids in trained and untrained young men. Am J Physiol-Endocrinol Metabol. 2000;279(4):E744‐E751. 10.1152/ajpendo.2000.279.4.E744 11001754

[50] Xiao D , Chang W. Phosphatidylserine in diabetes research. Mol Pharm. 2023;20(1):82‐89. 36480277 10.1021/acs.molpharmaceut.2c00707

[51] Li K , Wan B, Li S, et al Mitochondrial dysfunction in cardiovascular disease: towards exercise regulation of mitochondrial function. Front Physiol. 2023;14:1063556. 36744035 10.3389/fphys.2023.1063556 PMC9892907

[52] Dudek J . Role of cardiolipin in mitochondrial signaling pathways. Front Cell Dev Biol. 2017;5(SEP):90. 29034233 10.3389/fcell.2017.00090 PMC5626828

[53] El-Hafidi M , Correa F, Zazueta C. Mitochondrial dysfunction in metabolic and cardiovascular diseases associated with cardiolipin remodeling. Biochim Biophys Acta (BBA)—Mol Basis Dis. 2020;1866(6):165744. 10.1016/j.bbadis.2020.165744 32105822

