# Correction to: “Moderate-intensity Combined Training Induces Lipidomic Changes in Individuals With Obesity and Type 2 Diabetes”

**DOI:** 10.1210/clinem/dgae328

**Published:** 2024-05-31

In the above-named article by Duft RG, Bonfante ILP, Palma-Duran SA, Chacon-Mikahil MPT, Griffin JL, and Cavaglieri CR (*J Clin Endo Metab*. 2024; doi: 10.1210/clinem/dgae177), there were errors in the Disclosure and Acknowledgment sections.

In the originally published article, the Disclosure section read: “The authors have nothing to disclose.” In the corrected article, the Disclosure section reads: “Publication charges were paid by the UK Consortium for MetAbolic Phenotyping (MAP UK, - Medical Research Council (MR/S010483/1)).”

In the originally published article, the Acknowledgment section read: “The authors thank the volunteers for participating in the study.” In the corrected article, the Acknowledgment section reads: “The authors thank the volunteers for participating in the study. The authors thank the UK Consortium for MetAbolic Phenotyping (MAP UK, - Medical Research Council (MR/S010483/1)) for payment of publication fees.”

The article has been corrected online.

doi: 10.1210/clinem/dgae177

